# Study and use of the probiotic *Lactobacillus reuteri* in pigs: a review

**DOI:** 10.1186/s40104-015-0014-3

**Published:** 2015-04-09

**Authors:** Chengli Hou, Xiangfang Zeng, Fengjuan Yang, Hong Liu, Shiyan Qiao

**Affiliations:** State Key Laboratory of Animal Nutrition, China Agricultural University, Beijing, 100193 China

**Keywords:** Antibiotics, Application, *Lactobacillus reuteri*, Pigs, Probiotics

## Abstract

Probiotics are living microorganisms that provide a wide variety of health benefits to the host when ingested in adequate amounts. The bacterial strains most frequently used as probiotic agents are lactic acid bacteria, such as *Lactobacillus reuteri*, which is one of the few endogenous *Lactobacillus* species found in the gastrointestinal tract of vertebrates, including humans, rats, pigs and chickens. *L. reuteri* is one of the most well documented probiotic species and has been widely utilized as a probiotic in humans and animals for many years. Initially, *L. reuteri* was used in humans to reduce the incidence and the severity of diarrhea, prevent colic and necrotic enterocolitis, and maintain a functional mucosal barrier. As interest in alternatives to in-feed antibiotics has grown in recent years, some evidence has emerged that probiotics may promote growth, improve the efficiency of feed utilization, prevent diarrhea, and regulate the immune system in pigs. In this review, the characteristics of *L. reuteri* are described, in order to update the evidence on the efficacy of using *L. reuteri* in pigs.

## Introduction

Antibiotics are a common additive in livestock feed which have been widely used for growth promotion and prophylaxis purposes in farm animals during the past several decades [1]. However, antibiotic resistance is a looming public health crisis. The use of antibiotics as growth promoters has been forbidden in the European Union, Korea, and Japan. Other countries including the United States and China may ban the feeding of antibiotics within the next few years. As a result, there is increasing interest concerning alternatives to in-feed antibiotics, such as probiotics, prebiotics, plant products and organic acids in the livestock industry [2].

Probiotics are living microorganisms, which, when consumed in adequate amounts, can confer a health benefit to the host [3]. In farm animals, probiotics have been shown to promote growth, improve the efficiency of feed utilization, modulate the gastrointestinal ecosystem, stimulate the immune system and protect the host from gastrointestinal tract (GIT) diseases [4]. Therefore, probiotics provide a potential alternative strategy to in-feed antibiotics [5].

The properties of probiotics are strain-specific, and suitable probiotic strains for pigs are usually selected based on some criteria including pig origin, acid and bile tolerance, their ability to adhere to intestinal cells and to colonise the intestinal tract, production of antimicrobial substances, antibiotic resistance patterns, demonstrable efficacy and safety, and stability to the conditions used in industrial processes [6-8]. The organisms most frequently used as probiotic agents are lactic acid bacteria (LAB) [9], such as *Lactobacillus*, which are a normal inhabitant of the GIT [10]. *Lactobacillus reuteri* is one of the dominant species in the GIT of vertebrates such as humans, rats, pigs and chickens [11]. It is one of the most well documented probiotic species and has been widely utilized as a probiotic in humans and animals [12-15].

In recent years, numerous probiotic strains have been used in pig production. The application of probiotics provide a potential alternative strategy to the use of antibiotics. The aim of this review is to systematically review and update the evidence on the efficacy of using *L. reuteri* in pigs.

## Characteristics of *Lactobacillus reuteri*

*L. reuteri* is a heterofermentative bacterium, and is considered to be one of the few true autochthonous *Lactobacillus* species in humans and animals. Many researchers have already selected some specific *L. reuteri* strains isolated from human feces, breast milk, the human vagina, the human oral cavity, guinea pigs, rats, pigs, broilers and sourdough. There is now mounting evidence to show that selected *L. reuteri* strains have probiotic characteristics, and can provide health benefits to their hosts. We have constructed a summary table (Table 1), in order to provide an overview of the reported *L. reuteri* strains used as probiotics.

**Table 1:** **The strains and probiotic characteristics of reported** ***L. reuteri***

**Strain**	**Source**	**Characteristics**	**Reference**
*L. reuteri* I5007	piglets	strong adhesion, competitiveness against pathogens; improved pig performance, immune function and antioxidant status; alleviated the weaning stress syndrome; modulated gut microflora	[21,35,46-48,50,52]
*L. reuteri* NCIMB 30242	pig	improve gastrointestinal health; inhibite sterol absorption; increase mean circulating 25-hydroxyvitamin D	[61-63]
*L. reuteri* ATCC 53608	pig	recognize immunoglobulins	[24,41]
*L. reuteri* BSA131	pig	resistance to pH, oxgall and antibiotics, and antimicrobial activities against enteric pathogenic, improved pig performance	[12]
*L. reuteri* Pg4	broilers	tolerate acid and bile salts; inhibit pathogenic bacteria; adhere to intestinal epithelial cells; improved broiler performance	[17]
*L. reuteri* ATCC 55730	breast milk	colonize the intestinal tract; resistance to tetracycline and lincomycin; maintain intestinal health; prevent diarrhea; modulate the immune system; used in treatment of *Helicobacter pylori*	[13,14,64,65]
*L. reuteri* DSM 17938	ATCC 55730	a daughter strain derived from ATCC 55730 and has the same properties as ATCC 55730	[42]
*L. reuteri* ATCC PTA 4659	breast milk	partly prevent diet-induced obesity possibly via the mechanism of inducing liver expression of *Cpt1a*.	[66]
*L. reuteri* ATCC PTA 6475	breast milk	protect mice from disease manifestations of enterohemorrhagic *E. coli*	[67]
*L. reuteri* ATCC PTA 5289	oral cavity	improve oral malodour; reduce the number of selected periodontal pathogens in the subgingival microbiota	[68,69]
*L. reuteri* DPC16	human feces	produce reuterin	[29]
*L. reuteri* DSM 20016	human feces	produce reuterin	[30]
*L. reuteri* JCM 1112	human feces	produce reuterin and cobalamin	[31]
*L. reuteri* RC-14	human vagina	produce hydrogen peroxide; adhere to uroepithelial cells and inhibit uropathogens; modulate immunity	[28,70]
*L. reuteri* GMN-32	-	regulate blood glucose levels, protect cardiomyocytes and prevent diabetic cardiomyopathy in diabetes mellitus rats	[71]
*L. reuteri* DSMZ 17648	-	reduce the load of *Helicobacter pylori*	[72]
*L. reuteri* GMNL-263	-	ameliorate hepatic steatosis observed in high fructose treated rats; protect streptozotocin-induced diabetic rats from hyperglycemia-enhanced renal fibrosis	[73,74]
*L. reuteri* R2	-	strong inhibitory activity against the dermatophyte *Trichophyton tonsurans*	[75]
*L. reuteri* TD1	rat	not produce reuterin, exhibit a similar onset of type 1 diabetes	[76]
*L. reuteri* 100-23	rat	stimulate the development of regulatory T cells; transiently activates intestinal epithelial cells	[36,77]
*L. reuteri* BR11	guinea pig	unique antioxidant properties, show promise in the treatment of experimental colitis	[37]
*L. reuteri* CRL1098	sourdough	produce vitamin B_12_	[34]
*L. reuteri LTH2584*	sourdough	produce reutericyclin	[32]

Note: *L. reuteri* I5007, initially known as *L. fermentum* I5007; *L. reuteri* BR1, initially known as *L. fermentum* BR11; *L. reuteri* RC-14, initially known as *L. fermentum* RC-14.

### Probiotic properties

Probiotic bacteria encounter various stresses after ingestion by the host, including exposure to a low pH in the stomach and contact with bile in the small intestine. *L. reuteri* I5007, initially known as *Lactobacillus fermentum* I5007, was selected from over 7,000 native Lactobacilli colonies according to criteria including resistance to heat, low pH, copper, and bile salts, as well as storage stability and antagonism to pathogenic agents [16]. Other *L. reuteri* strains also show resistance to low pH and bile salts [12,17-19].

Adhesion of a probiotic strain to the host GIT is important for bacterial colonization, pathogen exclusion, and interaction with host cells for the protection of epithelial cells or immune modulation [20]. Several studies have demonstrated that *L. reuteri* have the capacity to colonize, and can adhere to mucin and intestinal epithelial cells [17,21-23]. *L. reuteri* I5007 shows strong adhesion to Caco-2 cells, IEC-6 cells, IPEC-J2 cells, and porcine intestinal mucus [15,21]. The possible mechanism for *L. reuteri* adherence and colonization involved in adhesion, has been linked to mucus-binding protein [24], surface protein [22], D-alanyl-LTA [25], exopolysaccharide [26], glucosyltransferase A and inulosucrase [27].

*L. reuteri* has been reported to produce a variety of antimicrobial substances such as lactic acid, hydrogen peroxide [28], reuterin [29-31], and reutericyclin [32], which have beneficial effects for the host organism. *L. reuteri* strains have been demonstrated to inhibit the *in vitro* growth of many enteric pathogens, including *Escherichia coli*, *Salmonella* Typhimurium, *Staphylococcus epidermidis*, *Staphylococcus aureus*, *Helicobacter pylori*, and rotavirus [12,19,33]. In addition, *L. reuteri* can produce vitamin B_12_ [31,34], and has the capacity to *de novo* synthesize L-lysine and folic acid based on a computer simulation model [15].

*L. reuteri* exhibited free radical-scavenging capacity *in vitro* [35], and encoded various antioxidant enzymes [15]. Studies in animals and humans have shown that oral administration of *L. reuteri* reduced the incidence and the severity of diarrhea, decreased visceral pain, prevented colic and necrotic enterocolitis, maintained a functional mucosal barrier, and induced colonization and immunomodulation [36-39].

### Safety and stability aspects

*L. reuteri* has the most extensive safety assessment record of any probiotic strain. A number of studies conducted both *in vivo* and *in vitro* indicate that *L. reutei* is safe for human consumption, even in large amounts [38,40]. However, as is the case for all other species of LAB, plasmids can be found in some strains of *L. reuteri* [15,41,42], and some of these plasmids have been shown to encode for antibiotic resistance genes [42]. According to the European Food Safety Authority, probiotics should not contain known antibiotic resistance traits. *L. reuteri* ATCC 55730 is a commercially available probiotic strain which has been found to carry potentially transferable resistance traits for tetracycline and lincomycin. Therefore, it has been replaced by *L. reuteri* DSM 17938, a strain where the two resistance plasmids have been removed without losing any probiotic characteristics [42].

Probiotic strains must be able to resist any adverse conditions encountered during industrial production in order to survive [43]. *L. reuteri* is sensitive to heat, and therefore, freeze-drying is commonly used for maintaining the stability of *L. reuteri*. Subjecting *L. reuteri* to a higher fermentation temperature (47°C) or a neutral pH (pH 6.7) has been shown to increase the survival of *L. reuteri* during subsequent freeze-drying [44].

### Strains

Not all *L. reuteri* strains are the same or provide a beneficial response, and the evolution of *L. reuteri* with vertebrates resulted in the emergence of host specialization [45]. Probiotic strains need to be carefully chosen, and evaluated for their safety and effectiveness using *in vitro* assays, animal models, and clinical trials. There are numerous strains of *L. reuteri*, which have some minor differences that make them unique (Table 1).

*L. reuteri* DSM 17938, *L. reuteri* NCIMB 30242, *L. reuteri* ATCC PTA 6475 which are of human origin are the most commonly used in dietary supplements and have been researched the most. In pigs, *L. reuteri* I5007, was isolated from the colonic mucosa of healthy weaning piglets, and has been demonstrated in several studies to have probiotic properties [15,21,35,46-49].

## Applications of probiotic *L. reuteri* for pigs

In pigs, the administration of *L. reuteri* has been shown to have beneficial effects on performance, prevention of diarrhea, stress relief, altered gut microbiota, and immunomodulation. The applications of *L. reuteri* for pigs are listed in Table 2. Noticeably, *L. reuteri* is mainly used in neonatal piglets and during the post-weaning period.

**Table 2:** **The application of probiotic** ***L. reuteri*** **in pigs**

**Strain**	**Dose**	**Animal**	**Significant results**	**Reference**
*L. reuteri* I5007	6 × 10^9^ CFU/d	newborn piglets	increased average daily gain; reduced diarrhea incidence; affected the colonic microbial communities , in particular, reduced numbers of *Clostridium* sp; reduced mRNA expression of IL-1β in the ileum	[46]
*L. reuteri* I5007	2× 10^9^ CFU/d	weaned pigs	increased weight gain and feed conversion; decreased the occurrence of diarrhea; enhanced T-cell differentiation and induced cytokine expression in the ileum	[47]
*L. reuteri* I5007	2× 10^9^ CFU/d	weaned pigs	had faster growth and higher feed intakes; improved the anti-oxidative defence system and alleviated damage caused by diquat	[50]
*L. reuteri* I5007	5.8 × 10^7^ CFU/g	weaned pigs	increased weight gain, feed intake and apparent crude protein digestibility; increased serum specific anti-OVA IgG level	[52]
*Lactobacilli* complex	10^5^ CFU/g	weaned pigs	increased weight gain and feed intake compared with carbadox; prevented diarrhea; decreased *E. coli* and aerobe counts, increased Lactobacilli and anaerobe counts in digesta and mucosa	[16]
*L. reuteri* BSA131	2× 10^6^, 2× 10^8^ CFU/g	weaned pigs	improved weight gain and feed conversion; decreased the number of enterobacteria in the feces	[12]
*L. reuteri* X-1	10^8^ CFU/g	weaned pigs	improved weight gain and feed conversion; decreased serum IgG and IgM concentrations, incresed serum DAO and D-lactate concentrations	[51]
*L. reuteri* I5007	1.02 × 10^8^ CFU/g	growing pigs	increased total antioxidant capacity	[35]
*L. reuteri* I5007	10^8^ CFU/g	weaned pigs	increaed weight gain, neither body weights nor weight gains differed between the *L. reuteri* and aureomycin groups; alleviated weaning stress syndrome	[48]
*L. reuteri* and *L. plantarum* complex	10^6^ CFU/g	weaned pigs	increased apparent total tract digestibility of nitrogen, gross energy, and fecal *Lactobacillus* concentration; decreased fecal gas emission, diarrhea score, and *E. coli* concentration	[78]
*L. reuteri* 3S7 and *L. plantarum* 4.1	10^10^ CFU/d	sows and piglets	were found in the faeces; decreased the population of *Enterobacteriaceae*; decreased β-glucuronidase activity of all pigs	[79]

Note: *L. reuteri* I5007 was initially known as *L. fermentum* I5007.

Lactobacilli complex including *L. gasseri*, *L. reuteri*, *L. acidophilus* and *L. fermentum* (renamed *L. reuteri* I5007).

### Improved performance

In the pig industry, the use of probiotics improves intestinal health which can improve pig performance. Supplementation of *L. reuteri* has resulted in improved growth and feed efficiency in neonatal and growing pigs. Liu *et al.* [46] reported that *L. reuteri* I5007 (6 × 10^9^ CFU/d) supplementation increased average daily gain (ADG) in formula-fed piglets. Wang *et al.* [47] found that administration of *L. reuteri* I5007 significantly increased weight gain and feed conversion compared with weaned pigs fed without *L. reuteri* I5007. Also, Wang *et al.* [50] reported that weaned piglets supplemented with *L. reuteri* had faster growth and higher feed intakes than unsupplemented piglets. However, feed conversion was unaffected by *L. reuteri* supplementation. In addition, Wang *et al.* [48] showed that dietary supplementation with *L. reuteri* or aureomycin significantly improved the performance of weanling piglets, and there was no difference between the two feed additives. Other studies using *L. reuteri* BSA131 tended to show improved ADG and feed conversion in weaned pigs [12]. Wang *et al*. [51] also reported that supplementation with *L. reuteri* X-1 increased ADG and feed conversion. Yu *et al.* [52] determined the influence of different levels of *L. reuteri* I5007 on performance, nutrient digestibility and immunity of weaned pigs. The results demonstrated that the ideal supplemental concentration of *L. reuteri* was 5.8 × 10^7^ CFU/g feed.

### Prevention of diarrhea

Diarrhea is one of the most frequent causes of heavy economic losses in swine operations [53]. The effect of *L. reuteri* against diarrhea in pigs was confirmed in several reports [16,46,47,54,55]. Diarrhea incidence was lower in piglets fed *L. reuteri* I5007 compared with a control [46]. Enterotoxigenic *E. coli* (ETEC) are a major cause of diarrhea in neonatal and weaned pigs [55]. Huang *et al*. [16] showed that a native Lactobacilli complex preparation (including *L. gasseri*, *L. reuteri*, *L. acidophilus* and *L. fermentum*) could effectively prevent weaning piglet diarrhea when administered before challenge with an *E. coli* solution (serovars K99, K88 and 987P at a ratio of 1:1:1). Wang *et al*. [47] reported that 12, 24, and 48 h after challenge, pigs challenged with *E. coli* had mild diarrhea and mild fecal scores. Supplementation of *L. reuteri* I5007 did not alleviate these effects. Only on day 10, did feeding *L. reuteri* I5007 decrease the occurrence of diarrhea. Chen *et al*. [54] demonstrated that reuteran produced by *L. reuteri* may prevent piglet diarrhea by reducing adhesion of ETEC K88.

### Alleviate stress

Pigs in industrial farming systems are frequently exposed to oxidative stress, which results in decreased performance and reduced immune function. *L. reuteri* has been shown to be effective in scavenging free radicals *in vitro*, and could be used to alleviate oxidative stress [35,37]. Wang *et al*. [35] reported that supplementation of *L. reuteri* I5007 improved the antioxidant status of growing-finishing pigs (from 50 to 90 kg) as evidenced by increased levels of antioxidant enzymes such as superoxide dismutase and glutathione peroxidase, and decreased levels of malondialdehyde. Wang *et al.* [50] determined the anti-oxidative effect of *L. reuteri* I5007 in weaning piglets using an oxidative stress model induced by diquat. Their results showed that diquat-injection decreased the performance of weaning pigs and increased plasma levels of cortisol, adrenaline, carbonyl and malondialdehyde. *L. reuteri* supplementation alleviated oxidative stress and enhanced the performance of weanling pigs.

Weaning is one of the most stressful periods that results in gastrointestinal, immunological, and behavioral changes [56]. Wang *et al*. [48] demonstrated that *L. reuteri* I5007 alleviated weaning stress syndrome by enhancing the levels of proteins involved in energy metabolism, lipid metabolism, cell structure and mobility, protein synthesis, and immune response, thereby facilitating cellular proliferation and depressing apoptosis.

### Modulation of gut microbiota

*L. reuteri* in neonatal piglets can be used to support the development of a stable microbiota, to stimulate the immune system and to prevent diarrheal diseases. During the weaning and post-weaning periods, *L. reuteri* is used in pigs to modulate the gastrointestinal microbiota as it aims to prevent post-weaning diarrhea and stimulate growth. Liu *et al*. [46] reported that *L. reuteri* I5007 plays a positive role in gut development in neonatal piglets by modulating the microbial composition and intestinal development, Denaturing gradient gel electrophoresis (DGGE) revealed that *L. reuteri* I5007 affected the colonic microbial communities on day 14 and, in particular, reduced numbers of *Clostridium* spp. In weaning pigs, administration of *L. reuteri* BSA131 decreased the number of enterobacteria in the feces [12]. Huang *et al*. [16] showed that a Lactobacilli compound (including *L. gasseri*, *L. reuteri*, *L. acidophilus* and *L. fermentum*) significantly decreased *E. coli* and aerobe counts, and increased Lactobacilli and anaerobe counts in the digesta and mucosa of most sections of the GIT compared with a control group. In addition, oral administration of *L. reuteri* I5007 not only increased the concentration of butyrate and other branched chain fatty acids but also decreased *Clostridium* strains accompanied by a lowered pH in the colonic digesta [46]. This indicates that administration of *L. reuteri* modulates gut microbiota, and thereby affected the microbial metabolites.

### Immunomodulation

Probiotics such as *L. reuteri* may stimulate or suppress innate immune responses via several mechanisms including modulation of pro-inflammatory cytokines. *L. reuteri* strains can be divided into two subsets, immunosuppressive (ATCC PTA 6475 and ATCC PTA 5289) and immunostimulatory strains (ATCC 55730 and CF48-3A), and each subset has potential therapeutic value [57]. The effects of *L. reuteri* on immunomodulation were documented in pigs. Wang *et al*. [47] reported that oral administration of *L. reuteri* I5007 could enhance T-cell differentiation and induce ileal cytokine expression, which suggests that this probiotic strain could modulate immune function in weaned piglets. Yu *et al*. [52] showed that *L. reuteri* I5007 supplementation increased serum specific anti-OVA IgG levels. In neonatal piglets, *L. reuteri* has been found to decrease the mRNA expression of IL-1β in the ileum [46]. Azevedo *et al*. [58] found that *L. reuteri* combined with *L. acidophilus* could help to maintain immunological homeostasis in neonatal gnotobiotic pigs infected with human rotavirus by regulating TGF-β production.

## Conclusions and perspectives

**Figure 1:**
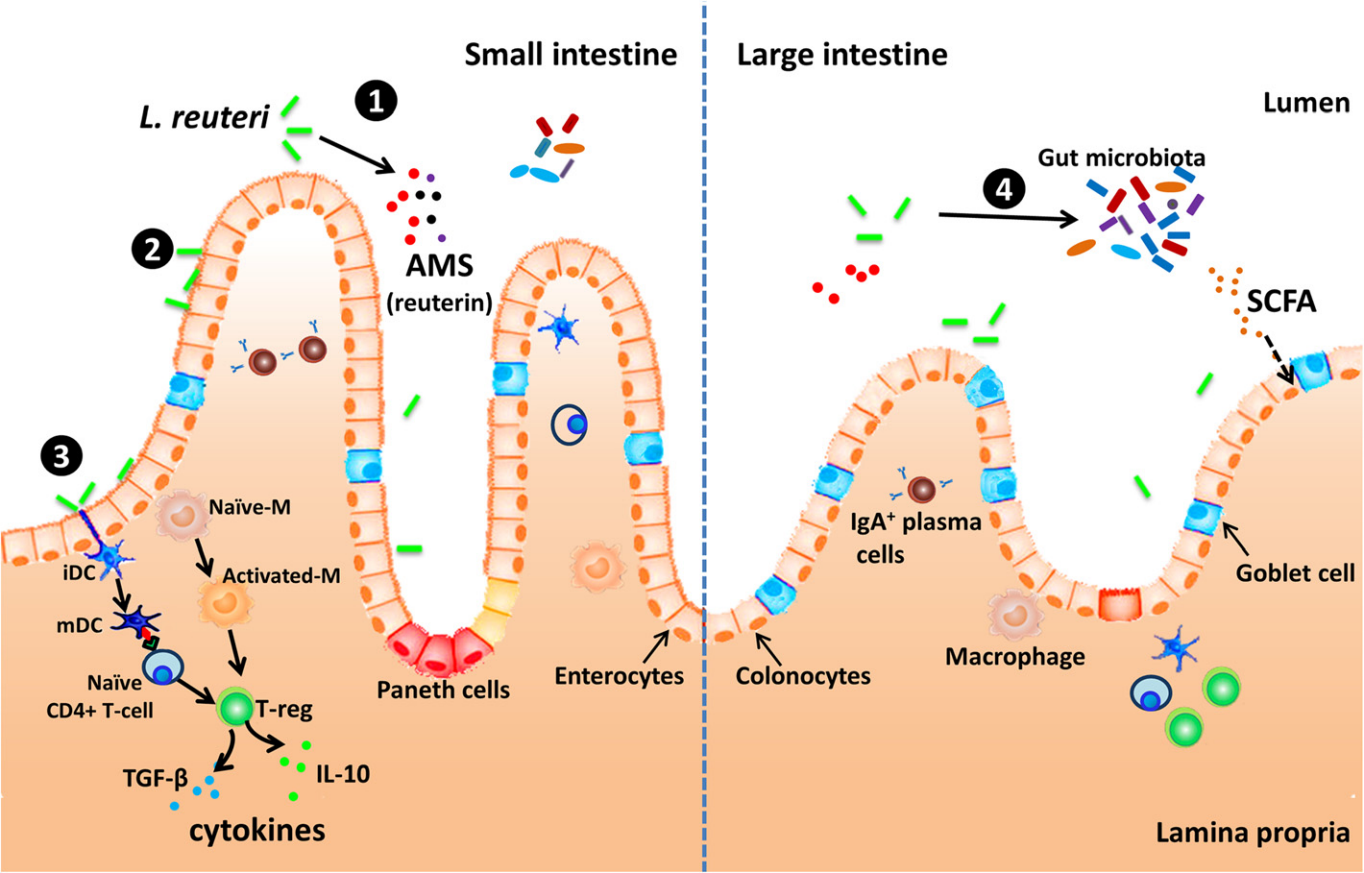
**Mechanisms of** ***L*** *.* ***reuteri*** **modulating in the gut.** ① *L. reuteri* can produce a variety of antimicrobial substances (AMS) such as lactic acid, and reuterin [28-30]. ② *L. reuteri* has the capacity to colonize, and can adhere to mucin and intestinal epithelial cells [17,21,22] . ③ *L. reuteri* has been shown to stimulate or suppress innate immune responses by affected the production of cytokines in macrophages (M), monocytes, and dendritic cells (DCs). The modulation of dendritic cells by *L. reuteri* has been shown to be mediated through dendritic cell-specific intercellular adhesion molecule-3-grabbing non-integrin (DC-SIGN) and promote development of regulatory T cells producing high amounts of interleukin-10 (IL-10) and transforming growth factor-β (TGF-β) [59,60]. ④ *L. reuteri* has been reported affected the colonic microbial communities and short chain fatty acid (SCFA) concentration [46]. Please see text for details and references.

In conclusion, *L. reuteri* is a probiotic bacteria that is one of the few true autochthonous *Lactobacillus* species. Numerous studies have demonstrated that they can positively improve performance, prevent diarrhea, alleviate stress, alter gastrointestinal microbiota, regulate the immune system, and thereby improve pig performance and health. The beneficial effects of *L. reuteri* in pigs have been related to different modes of action. The improvements in pig performance of supplemental *L. reuteri* are mostly due to the fact that *L. reuteri* has the ability to colonize the GIT, produce antimicrobial substances and stimulate the intestinal immune system (Figure 1), thereby promoting nutrient metabolism and improve health. However, a clear mode of action has yet to be described. It appears from the data presented that the benefical effects of *L. reuteri* are strain specific. It will be important to select more powerful or targeted strains. Unfortunately, the viability of *L. reuteri* is a key criteria for developing *L. reuteri* products. To expand the probiotic *L. reuteri* application in pigs, care must be taken during processing techniques such as microencapsulation to maintain bacterial stability.

Pig husbandry has entered an era when the use of antibiotics is increasingly unwelcomed. Probiotics, which are a potential alternative to in feed antibiotics, can expect a promising future. Besides selection of excellent strains and improved processing techniques, more research, especially in the form of well-designed animal trials, is needed to evaluate the efficacy of *L. reuteri*. More studies are also needed to explore the mechanisms of action of *L. reuteri* in pigs. An important fact is that *L. reuteri* added to pig diets may potentially help improve performance. With evolving knowledge, effective use of *L. reuteri* will be possible in the future.
